# Supplementary material for: The interactome of intact mitochondria by cross-linking mass spectrometry provides evidence for coexisting respiratory supercomplexes
Source: Mol Cell Proteomics. 2017 Dec 8;17(2):216–32. doi: 10.1074/mcp.RA117.000470 (PMC5795388; doi:10.1074/mcp.RA117.000470)
Supplement: Supplemental Data [file supp_RA117.000470_133922_0_supp_23978_fzffwf.zip › spectra_annotation/mito_spectra_annotation/72-1-9-1-35-1.pdf]

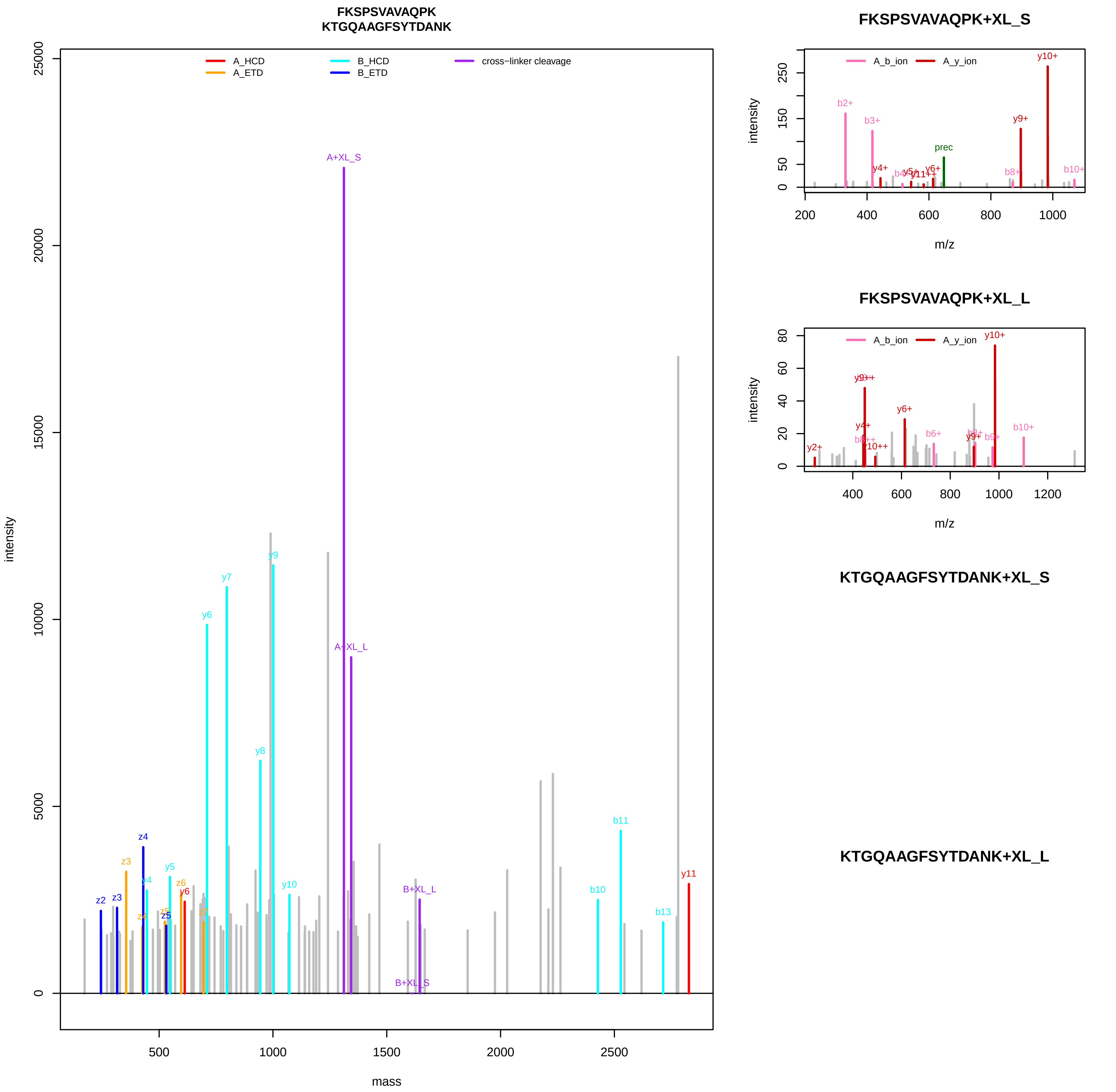
FKSPSVAVAQPK
KTGQAAGFSYTDANK
A_HCD
A_ETD
B_HCD
B_ETD
cross−linker cleavage
intensity
mass
A+XL_S
A+XL_L
B+XL_L
B+XL_S
y6
y7
y8
y9
y10
y11
b10
b11
b13
z2
z3
z4
z5
z6
y4
y5
FKSPSVAVAQPK+XL_S
A_b_ion
A_y_ion
b2+
b3+
y4+
y9+
y10+
prec
b8+
b10+
m/z
FKSPSVAVAQPK+XL_L
y2+
y6+
b6+
y9+
b9+
KTGQAAGFSYTDANK+XL_S
KTGQAAGFSYTDANK+XL_L
